# Supplementary material for: Navigation-grade interferometric air-core antiresonant fibre optic gyroscope with enhanced thermal stability
Source: Nat Commun. 2025 Apr 14;16:3449. doi: 10.1038/s41467-025-58381-6 (PMC11997165; doi:10.1038/s41467-025-58381-6)
Supplement: Supplementary file 1 — Supplementary Information [file 41467_2025_58381_MOESM1_ESM.pdf]

Supplementary Materials

Navigation-grade interferometric air-core anti-resonant fibre optic gyroscope with enhanced thermal stability

Maochun Li[1,†], Yizhi Sun[2,3,5†], Shoufei Gao[2,3,5†], Xiaoming Zhao[1,*], Fei Hui[1], Wei Luo[1], Qingbo Hu[2,3], Hao Chen[2,3], Helin Wu[2,3], Yingying Wang[2,3,5], Miao Yan[1,4] & Wei Ding[2,3,5,6*]

[1]Tianjin Key Laboratory of Quantum Precision Measurement Technology, Tianjin Navigation Instruments Research Institute, Tianjin 300131, China

[2]Guangdong Provincial Key Laboratory of Optical Fiber Sensing and Communication, Institute of Photonics Technology, Jinan University, Guangzhou 510632, China

[3]College of Physics & Optoelectronic Engineering, Jinan University, Guangzhou 510632, China

[4]School of Mechanical Engineering, Nanjing University of Science and Technology, Nanjing 210094, China

[5]Linfiber Technology (Nantong) Co., Ltd. Jiangsu 226010, China

[6]Pengcheng Laboratory, Shenzhen 518055, China

[†]These authors contributed equally to this work.

*Corresponding authors: dingwei@jnu.edu.cn; tjhhyq@yeah.net

Contents

S1. Testing and calculation of fibre characteristics **3**

S1.1 Higher-Order Modes 3

S1.2 Shupe Constant 3

S1.3 Linear Polarization Purity 4

S1.4 Fibre Stiffness 4

S2. Modeling and measurement of polarization properties **5**

S2.1 Modeling 5

S2.2 Crossed-Polarizer Transmission Spectra 7

S2.3 Principal Axis Angle Variations of Our four-tube *t*DNANF 8

S3. Manufacture and test of the four-tube *t*DNANF gyro **9**

S3.1 Symmetrical Quadrupolar Fibre Winding 9

S3.2 Fibre-to-Chip Direct Coupling 10

S3.3 Overall Dimensions of the four-tube *t*DNANF Gyro 10

S3.4 FOG Static Performance Test 11

S3.5 FOG Thermal Excursion Measurement 11

S3.6 FOG Wide-Temperature Scale Factor Measurement 12

References and notes **13**

S1. Testing and calculation of fibre characteristics

S1.1 Higher-Order Modes

The relative amount of power carried by higher-order modes (HOMs) in NANFs is measured using the spatially and spectrally (S^2) imaging technique[1,2]. As depicted in Fig. S1, the setup comprises a tunable laser source (TLS, Santec TSL-550A, 1490 nm-1640 nm) and a CCD camera (WiDy Sens 320, NIT). The fibre under test (FUT) is a 510 m four-tube *t*DNANF used for the FOG. Light in the *t*DNANF is launched from standard single mode fibre (SSMF) by end-fire coupling. As the wavelength sweeps across a range of 2.5 nm with a step of 1 pm, the CCD camera records the spatial distributions of the transmitted light concurrently. Subsequently, the multipath interference (MPI) value, defined as the power ratio of the relevant modes at the output, can be calculated after Fourier analysis.

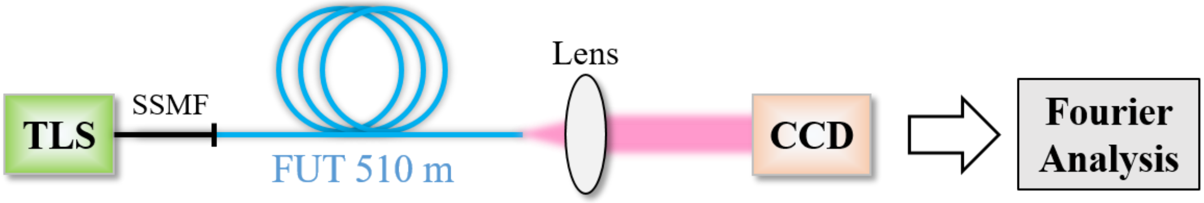

Fig. S1. Setup for S^2-imaging measurement.

S1.2 Shupe Constant

The thermal sensitivity of the accumulated phase (φ) of light propagating through a fibre, referred to as Shupe constant (S), can be expressed as[3]

$$S = \frac{1}{\varphi}\frac{d\varphi}{dT} = \frac{1}{L}\frac{dL}{dT} + \frac{1}{n_{eff}}\frac{dn_{eff}}{dT}, \tag{S1}$$

where n_{eff} is the effective refractive index, λ is the wavelength in vacuum, L is the fibre length, and T is the temperature.

Figure S2 shows the setup of a fibre Mach–Zehnder interferometer used in this work. A narrow linewidth laser (TLS, Santec TSL-550A, @1550 nm) with high frequency stability is employed. In the signal arm, the FUT is coiled with a radius of 6 cm and placed in a homemade thermal chamber alongside two thermometers (resolution of 0.1 °C). Both ends of the FUT are spliced to a piece of SSMF. The length difference between SSMFs in the signal and the reference arms is less than 2 cm to minimize environmental temperature induced phase drift. After a 3×3 fibre optic coupler, the interferograms are acquired in real-time by three InGaAs photodetectors (Thorlabs, PDA015C2) connected to a digital data acquisition card. Then, the thermally induced optical phase change over the FUT can be retrieved.

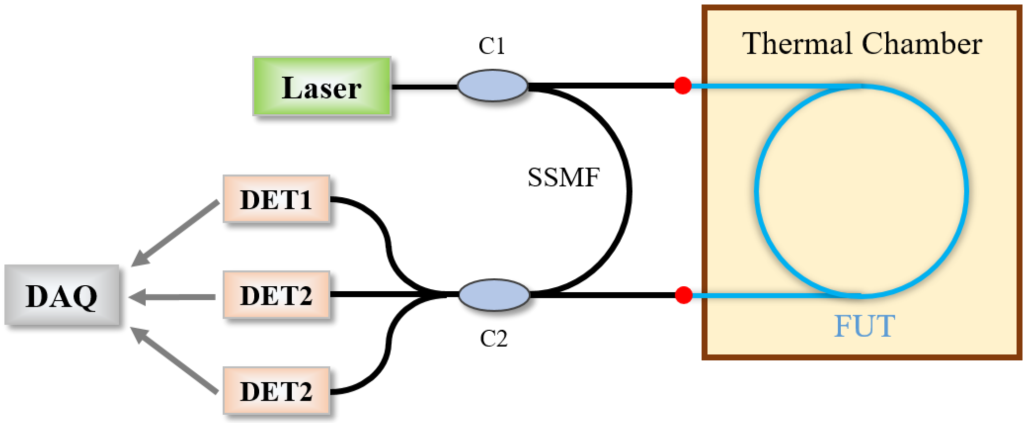

Fig. S2. Setup for Shupe constant measurement. C1, 2×2 fibre optic coupler (50:50); C2, 3×3 fibre optic coupler (33:33:33); Red points, fusion splices between SSMF and *t*DNANF; DET1-3, photodetectors; DAQ, data acquisition card.

S1.3 Linear Polarization Purity

To measure the polarization property of our *t*DNANF, a polarization measurement setup was built using an SC, two calcite polarizers (P1/P2), two achromatic half wave plates (HWP1/HWP2), and an OSA (Fig. S3). The FUT is coiled with a radius of 6 cm and placed in the thermal chamber. The input and output polarization states of the FUT are tuned by rotating HWP1 and HWP2, respectively.

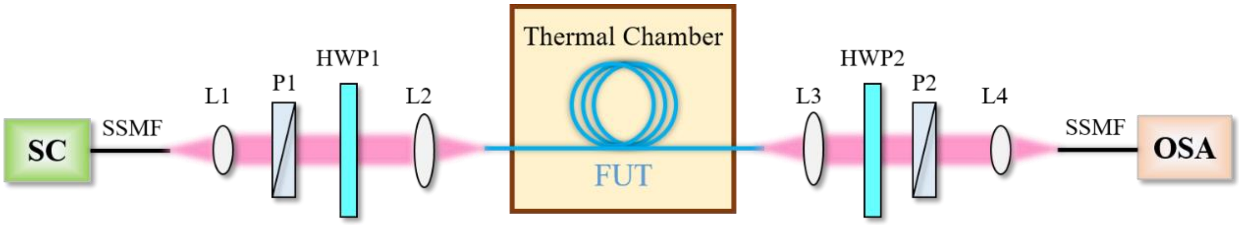

Fig. S3. Setup for linear polarization measurement. SC, supercontinuum source; L1-L4, lens; P1/P2, polarizers; HWP1/HWP2, half wave plates; OSA, optical spectrum analyzer.

S1.4 Fibre Stiffness

Stiffness is a measure of mechanical resistance to the longitudinal strain applied to a fibre, defined as the product of Young's modulus and the cross-sectional area. Considering the double acrylate coatings typically used for protecting optical fibers and using the material parameters from Ref. [4], Fig. S4 illustrates and compares the geometries of our four-tube *t*DNANF and the control PM-SCF. The stiffnesses $E_{silica} \cdot A_{silica} + E_{coating}^{(inner)} \cdot A_{coating}^{(inner)} + E_{coating}^{(outer)} \cdot A_{coating}^{(outer)}$ of the two fibres exhibit a ratio of 7.07:1. In this analysis, we consider room temperature and the representative Young's moduli: E_{silica} = 72.5 GPa, $E_{coating}$ (inner layer) = 158 MPa, and $E_{coating}$ (outer layer) = 780 MPa.

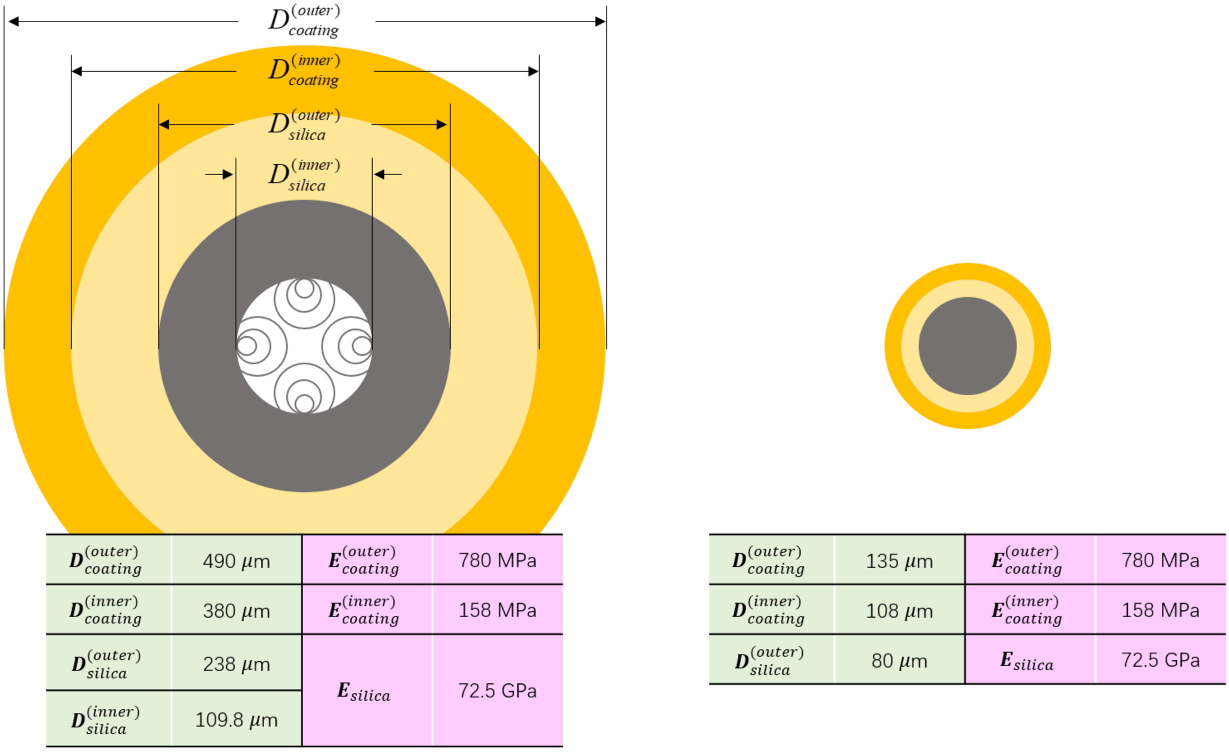

$D^{(outer)}_{coating}$	490 μm	$E^{(outer)}_{coating}$	780 MPa
$D^{(inner)}_{coating}$	380 μm	$E^{(inner)}_{coating}$	158 MPa
$D^{(outer)}_{silica}$	238 μm	E_{silica}	72.5 GPa
$D^{(inner)}_{silica}$	109.8 μm		

$D^{(outer)}_{coating}$	135 μm	$E^{(outer)}_{coating}$	780 MPa
$D^{(inner)}_{coating}$	108 μm	$E^{(inner)}_{coating}$	158 MPa
$D^{(outer)}_{silica}$	80 μm	E_{silica}	72.5 GPa

Fig. S4. Cross-sectional geometries of the four-tube *t*DNANF (left) and the control polarization-maintaining solid-core fibre (right), consisting of silica glass (shown in grey), soft acrylate inner coatings (light yellow), and hard outer coatings (dark yellow).

S2. Modeling and measurement of polarization properties

S2.1 Modeling

To facilitate the analysis of the polarization measurement results, it is imperative to model the birefringence properties of an AR-HCF, which are primarily dictated by two factors. The first one is intrinsic, related to the microstructure of an AR-HCF, including the ellipticity of the core and the non-uniformity of the membrane thickness[5]. The second factor is extrinsic, including macro-/micro-bending and twisting. As a result, an AR-HCF can be treated as a cascade of wave-plates (see Fig. S5), and each wave-plate possesses the wavelength-dependent principal axis angle and phase retardance of its own. When an AR-HCF is placed stably and under static temperature, the entire AR-HCF can be equivalently represented as a wavelength-dependent wave plate.

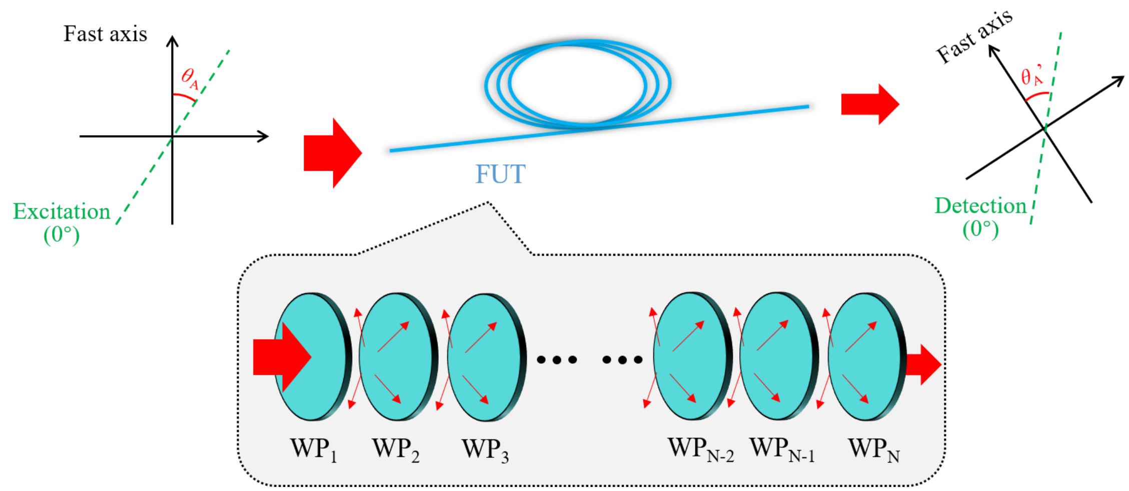

Fig. S5. Schematic of AR-HCF as a birefringent device and the crossed-polarizer transmission. WP: ideal wave plate.

During measurement, (0°/0°) refers to the situation where the excitation and the detection polarization angles are aligned to the input and the output principal axes by rotating the two HWPs, respectively. As shown in Fig. S5, θ_A is the angle between the excitation polarization and the fast axis, and θ'_A is the angle between the detection polarization and the fast axis. Note that both these angles are wavelength dependent and can only be zero at certain wavelengths. By rotating the two HWPs, the normalized transmission spectra can be expressed as

$$\begin{cases} I_{0^\circ/0^\circ}(\lambda) = \cos\left[\theta_A(\lambda) - \theta_A'(\lambda)\right]^2 - \frac{1}{2}\sin\left(2\theta_A(\lambda)\right)\cdot\sin\left(2\theta_A'(\lambda)\right)\cdot\left(1-\Delta\cdot\cos\left(\phi(\lambda)\right)\right) \\ I_{0^\circ/90^\circ}(\lambda) = \sin\left[\theta_A(\lambda) - \theta_A'(\lambda)\right]^2 + \frac{1}{2}\sin\left(2\theta_A(\lambda)\right)\cdot\sin\left(2\theta_A'(\lambda)\right)\cdot\left(1-\Delta\cdot\cos\left(\phi(\lambda)\right)\right) \\ I_{45^\circ/45^\circ}(\lambda) = \cos\left[\theta_A(\lambda) - \theta_A'(\lambda)\right]^2 - \frac{1}{2}\cos\left(2\theta_A(\lambda)\right)\cdot\cos\left(2\theta_A'(\lambda)\right)\cdot\left(1-\Delta\cdot\cos\left(\phi(\lambda)\right)\right) \\ I_{45^\circ/135^\circ}(\lambda) = \sin\left[\theta_A(\lambda) - \theta_A'(\lambda)\right]^2 + \frac{1}{2}\cos\left(2\theta_A(\lambda)\right)\cdot\cos\left(2\theta_A'(\lambda)\right)\cdot\left(1-\Delta\cdot\cos\left(\phi(\lambda)\right)\right) \end{cases}, \quad \text{(S2)}$$

where $\phi(\lambda)$ is the wavelength-dependent phase retardance accumulated between the two principal axes, whose spectrum can derive the group birefringence. $0 < \Delta < 1$ stands for an empirical correction factor to account for the lossy coupling between adjacent fibre segments or the 'wave-plates' outlined in Fig. S5. For the excitation and the detection polarization angles, it should be stressed that $\theta_A(\lambda)$ need not equal $\theta'_A(\lambda)$ across the whole transmission wavelength region of AR-HCF, spanning hundreds of nanometers. It is this inequality that brings about difficulties in calculating principal axis angles by using Eq. (S2).

In our measurement, we first adjust the two HWPs to obtain the minimum transmission power at a single wavelength (λ_0, i.e., 1545 nm in this work), which corresponds to a crossed-polarizer measurement (0°/90°) at $\theta_A(\lambda_0) = \theta'_A(\lambda_0) = 0$. In proximity to λ_0 (1525 - 1565 nm in this work), we

hypothesize $\theta_A(\lambda) \approx \theta'_A(\lambda)$ and therefore simplify Eq. (S2) to a form similar to the expression in Ref. [6]

$$\begin{cases} I_{0^\circ/0^\circ}(\lambda) = 1 - \frac{1}{2}\sin^2\left(2\theta_A(\lambda)\right)\cdot\left(1 - \Delta\cdot\cos\left(\phi(\lambda)\right)\right) \\ I_{0^\circ/90^\circ}(\lambda) = \frac{1}{2}\sin^2\left(2\theta_A(\lambda)\right)\cdot\left(1 - \Delta\cdot\cos\left(\phi(\lambda)\right)\right) \\ I_{45^\circ/45^\circ}(\lambda) = 1 - \frac{1}{2}\cos^2\left(2\theta_A(\lambda)\right)\cdot\left(1 - \Delta\cdot\cos\left(\phi(\lambda)\right)\right) \\ I_{45^\circ/135^\circ}(\lambda) = \frac{1}{2}\cos^2\left(2\theta_A(\lambda)\right)\cdot\left(1 - \Delta\cdot\cos\left(\phi(\lambda)\right)\right) \end{cases}. \quad \text{(S3)}$$

After acquiring the transmission spectra in the configurations of 0°/0°, 0°/90°, 45°/45°, 45°/135°, the wavelength dependences of $\theta_A(\lambda)$ [$\approx \theta'_A(\lambda)$] can be derived.

Based on the measured $\theta_A(\lambda)$, the broadband PER can be estimated by the following calculation when the input power spectrum is assumed to be uniform from λ_1 to λ_2

$$\begin{aligned} PER(dB) &= 10\times\log\frac{\int_{\lambda_1}^{\lambda_2} I_{0^\circ/0^\circ}(\lambda)d\lambda}{\int_{\lambda_1}^{\lambda_2} I_{0^\circ/90^\circ}(\lambda)d\lambda} \\ &= 10\times\log\frac{\int_{\lambda_1}^{\lambda_2}\left[1 - \frac{1}{2}\sin^2\left(2\theta_A(\lambda)\right)\cdot\left(1 - \Delta\cos\left(\phi(\lambda)\right)\right)\right]d\lambda}{\int_{\lambda_1}^{\lambda_2}\left[\frac{1}{2}\sin^2\left(2\theta_A(\lambda)\right)\cdot\left(1 - \Delta\cos\left(\phi(\lambda)\right)\right)\right]d\lambda}, \end{aligned} \quad \text{(S4)}$$

where λ_1 is 1525 nm and λ_2 is 1565 nm, respectively. Considering group birefringence at the level of 10^{-6} to 10^{-5}, the phase retardance $\phi(\lambda)$ can vary tens of 2π from 1525 nm to 1565 nm with the fibre length to be hundreds of meters. Therefore, the integrals in Eq. S5 can be simplified as

$$PER(dB) \approx 10\times\log\frac{\int_{\lambda_1}^{\lambda_2}\left[1 - \frac{1}{2}\sin^2\left(2\theta_A(\lambda)\right)\right]d\lambda}{\int_{\lambda_1}^{\lambda_2}\left[\frac{1}{2}\sin^2\left(2\theta_A(\lambda)\right)\right]d\lambda}. \quad \text{(S5)}$$

S2.2 Crossed-Polarizer Transmission Spectra

Utilizing the polarization measurement setup shown in Fig. S3, the transmission spectrum of the fibre can be obtained when the input/output linear polarization angle are rotated to 0°/0°, 0°/90°, and 45°/135°, respectively. Fig. S6(a) provides a typical result when a 3 m four-tube *t*DNANF is coiled with R_b = 6 cm at room temperature. Across the broad wavelength range of the transmission window of this fibre, the minimum transmission is only seen at ~1550 nm, where $\theta_A = \theta'_A = 0$. Within the wavelength range near 1550 nm, the principal axis angle slightly changes and $\theta_A \approx \theta'_A$

[the orange region in Fig. S6(a)], and Eq. (S3) can be used to retrieve θ_A. When the wavelength deviates far from 1550 nm [the green region in Fig. S6(a)], Eq. (S3) is no longer applicable because $\theta_A \neq \theta'_A$.

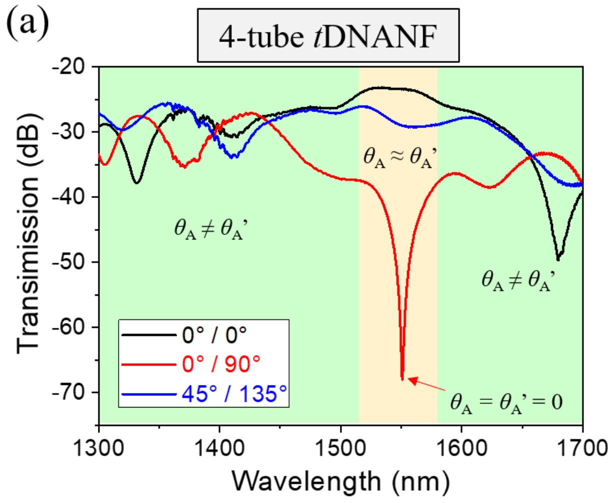

Fig. S6. Crossed-polarizer transmission spectra through 3 m (a) four-tube *t*DNANF and (b) five-tube NANF with R_b = 6 cm, respectively.

On the other hand, when considering a five-tube NANF (as mentioned in Fig. 2 of the main text), small-θ_A regions can hardly be found, as depicted in Fig. S6(b). The crossed-polarizer transmission spectra imply a rapid principal axis angle variation with wavelength. It seems that a 10^{-7} level of (low) birefringence cannot diminish the wavelength dependence of the principal axis angle. As a result, broadband PER will remain at a low level in this structure of ARFs.

S2.3 Principal Axis Angle Variations of Our four-tube *t*DNANF

Using crossed-polarizer transmission spectrum measurements and the model described in Eq. (S3), we tested our four-tube *t*DNANF under varying lengths and bend radii. As shown in Fig. S7, the measured principal axis offsets change under different conditions, indicating that imperfections in winding—such as twisting and stress— can slightly affect the principal axis. These imperfections are more challenging to eliminate in longer fibres. Nevertheless, within the spectral region of interest (1525-1565 nm), the variation remains relatively small, maintaining a broadband PER >20 dB. We attribute this performance primarily to the modest yet significant birefringence (on the order of 10^{-6}) in our four-tube *t*DNANF.

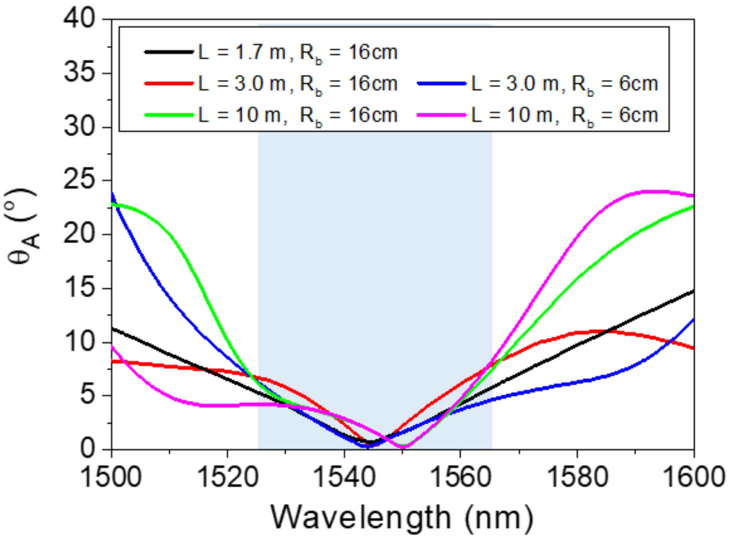

Fig. S7. Measured principal axis offsets relative to the orientations under different fibre lengths and bend radii at room temperatures.

S3. Manufacture and test of the four-tube *t*DNANF gyro

S3.1 Symmetrical Quadrupolar Fibre Winding

During the symmetrical quadrupolar fibre winding, on-line transmission loss is monitored to adjust the tension in time, thereby minimizing damage to the fibre. As shown in Fig. S8(a), an ASE source of 40 nm bandwidth and a power meter are connected to the two ends of the four-tube *t*DNANF rotating with fibre suppliers to provide for on-line transmission loss monitoring. The quadrupolar winding method, where the fibre layer order is reversed pair by pair (as shown in Fig. s8(b)), can greatly mitigate the Shupe effect of the whole coil.

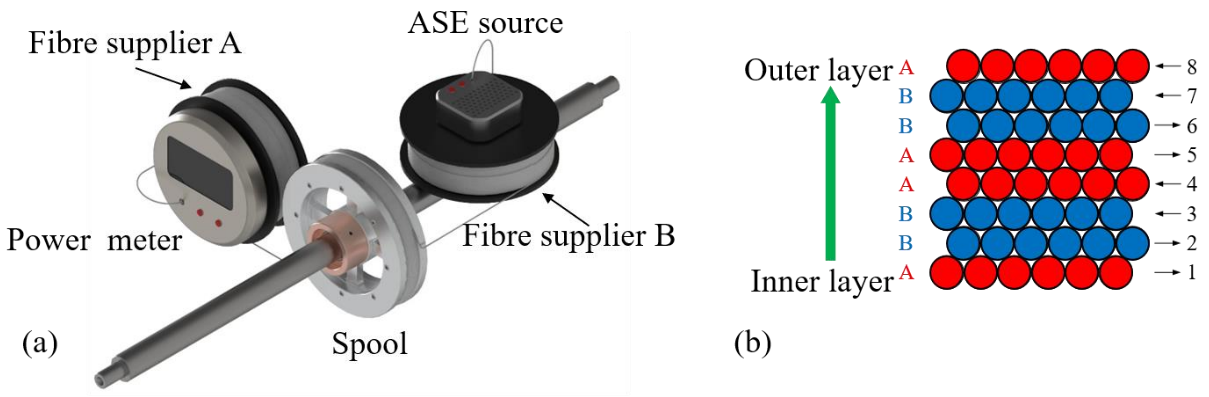

Fig. S8. Schematic of symmetrical quadrupolar fibre winding. (a) The on-line transmission loss monitoring setup. (b) Section view of symmetrical quadrupolar winding.

S3.2 Fibre-to-Chip Direct Coupling

On the fibre-to-chip direct coupling platform shown in Fig. S9, the four-tube *t*DNANF coil ends are directly connected to the multi-function integrated optics chip (MIOC). This operating platform consists of a set of motion control systems, a power meter, an ER meter, and other components.

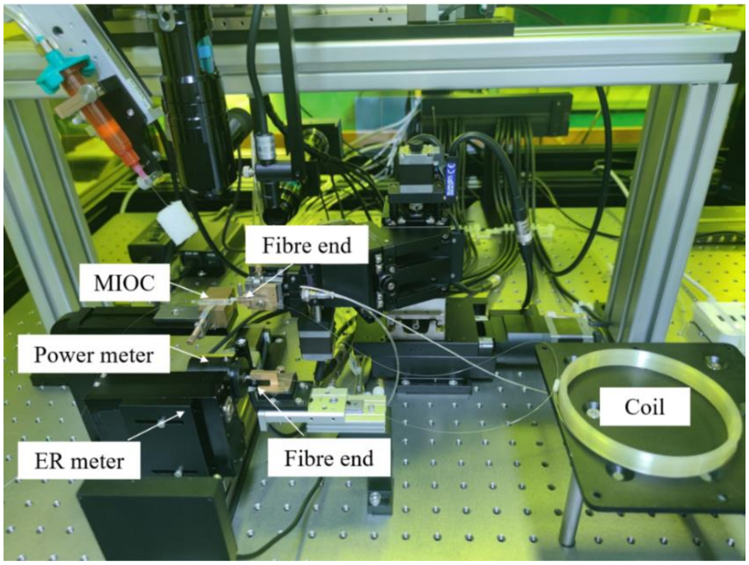

Fig. S9. Fibre-to-chip direct coupling assembly platform.

S3.3 Overall Dimensions of the four-tube *t*DNANF Gyro

The photograph of the four-tube *t*DNANF gyro is illustrated in Fig. S10, which adopts optic-electronic separation structure design. The photonic subsystem consists of a fibre coil and an MIOC, whose diameter and height are 156 mm and 20 mm, respectively. The electronic subsystem, with a size of 97.5×97.5×35 mm^3, consists of an ASE, a PMFC, a PD, and an electronics package.

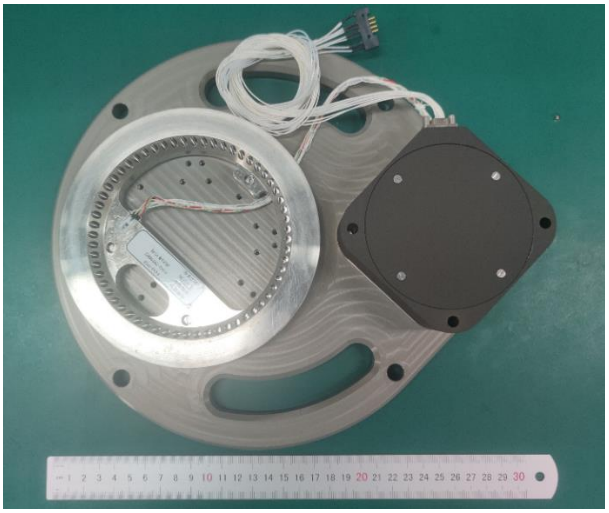

Fig. S10. Photograph of the four-tube *t*DNANF gyro.

S3.4 FOG Static Performance Test

The four-tube *t*DNANF gyro is mounted on a stable pier (as shown in Fig S11) with an input rotation rate only a fraction of Earth's rate, and the output data of the gyro are stored in a computer. At such low and stable input rate, any drift or variation can be attributed to random noise (ARW) over short integration times or bias stability/instability (BS/BI) over longer integration times.

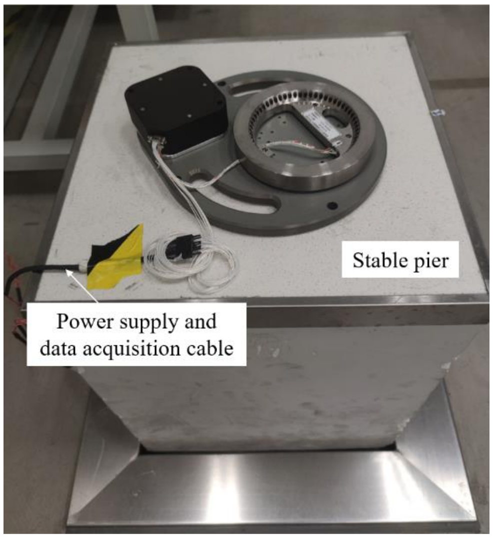

Fig. S11. Static performance test setup.

S3.5 FOG Thermal Excursion Measurement

The four-tube *t*DNANF gyro is stably placed on a vibration isolation base inside a temperature chamber (as shown in Fig. S12). The thermally induced rotation shift is measured at various temperature change rates across different temperature ranges.

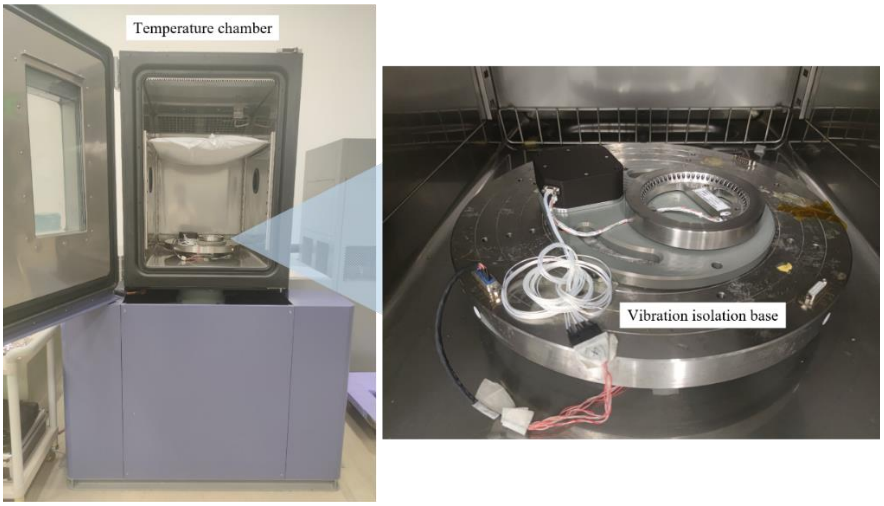

Fig. S12. Thermal excursion measurement setup.

S3.6 FOG Wide-Temperature Scale Factor Measurement

Under a nominal temperature change rate of 1 °C per minute, spanning a temperature range from -40 °C to 60 °C, a total of seven scale factor tests were conducted on the four-tube *t*DNANF gyro. Each test was carried out over a 10-minute interval, with rotation rates set to ±0.2°/s, ±0.5 °/s, ±1 °/s, ±2 °/s, ±5 °/s, ±10 °/s, ±20 °/s, ±50 °/s, ±100 °/s, ±200 °/s, ±300 °/s, and ±400 °/s, along with a 40 second dwell time for each rate. The rotation rates were systematically increased from lower to higher values, beginning with forward rotation followed by reverse rotation.

Figure S13 presents the results of these seven scale factor measurements across the wide temperature range for the four-tube *t*DNANF gyro. Specifically, Fig. S13a shows the gyro outputs corresponding to the various rotation rates, Fig. S13b illustrates the gyro temperature changes during the tests, and Fig. S13c displays the linear regression analysis of the seven measurement sets. The analysis reveals a scale factor repeatability of 11.1 ppm, a maximum scale factor nonlinearity of 3.2 ppm, and a maximum scale factor asymmetry of 6.1 ppm. These results confirm the gyro's excellent scale factor characteristics across the tested temperature range, ensuring that variations in the scale factor on the order of 10 ppm do not significantly impact the thermal sensitivity experiments.

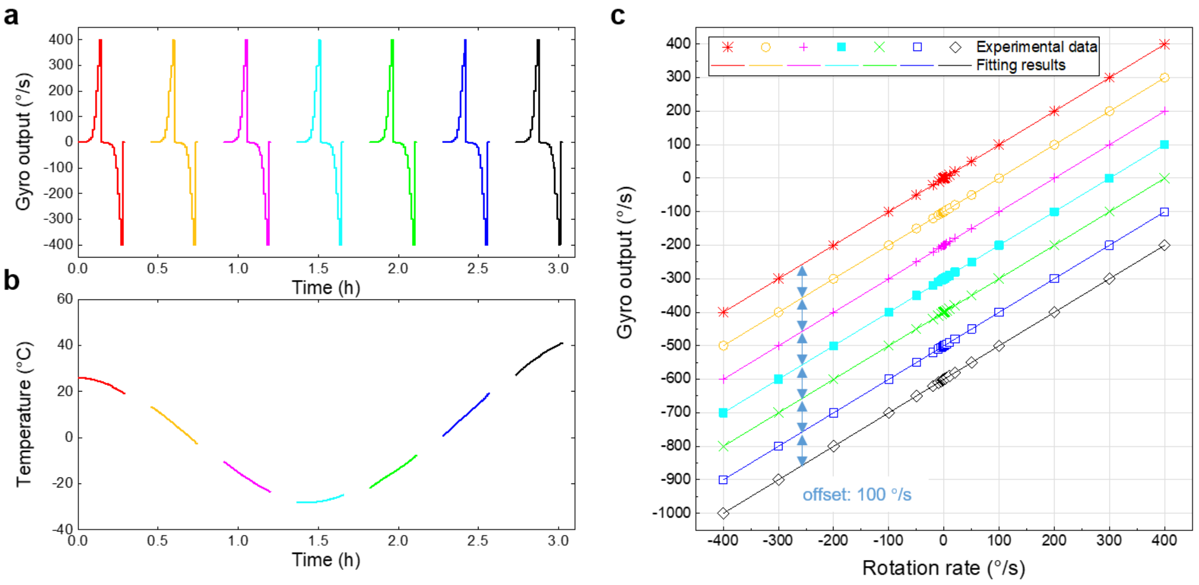

Fig. S13. Wide-Temperature Scale Factor Measurement. (a) Gyro outputs corresponding to different rotation rates. (b) Gyro temperature changes during measurements. (c) Rotation measurement results for the seven test sets. (*Note: A 100 °/s offset was artificially added between each set for clarity purposes.*)

References and notes

1. J. W. Nicholson, A. D. Yablon, S. Ramachandran & S. Ghalmi, "Spatially and spectrally resolved imaging of modal content in large-mode-area fibers," *Opt. Express* **16**, 7233–7243 (2008).
2. D. R. Gray, S. R. Sandoghchi, N. V. Wheeler, N. K. Baddela, G. T. Jasion, M. N. Petrovich, F. Poletti & D. J. Richardson, "Accurate calibration of S^2 and interferometry based multimode fiber characterization techniques," *Opt. Express* **23**, 10540–10552 (2015).
3. V. Dangui, H. K. Kim, M. J. F. Digonnet & G. S. Kino, "Phase sensitivity to temperature of the fundamental mode in air-guiding photonicbandgap fibers," *Opt. Express* **13**, 6669–6684 (2005).
4. B. Shi, H. Sakr, J. Hayes, X. Wei, E. Numkam Fokoua, M. Ding, Z. Feng, G. Marra, F. Poletti, D. J. Richardson, & R. Slavík, "Thinly coated hollow core fiber for improved thermal phase-stability performance," *Opt. Lett.* **46**, 5177-5180 (2021).
5. W. Ding & Y. Wang, "Hybrid transmission bands and large birefringence in hollow-core anti-resonant fibers," *Opt. Express* **23**, 21165–21174 (2015).
6. A. Taranta, E. Numkam Fokoua, S. Abokhamis Mousavi, J. R. Hayes, T. D. Bradley, G. T. Jasion & F. Poletti, "Exceptional polarization purity in antiresonant hollow-core optical fibres," *Nat. Photon.* **14**, 504–510 (2020).
